# Pyroptosis and chemical classification of pyroptotic agents

**DOI:** 10.1007/s11030-024-10987-6

**Published:** 2024-09-24

**Authors:** Mohammed A. Hara, Mohamed Ramadan, Mohammed K. Abdelhameid, Ehab S. Taher, Khaled O. Mohamed

**Affiliations:** 1 Pharmaceutical Organic Chemistry Department, Faculty of Pharmacy, Al Azhar University (Assiut), Assiut, 71524 Egypt; 2 Pharmaceutical Organic Chemistry Department, Faculty of Pharmacy, Cairo University, Cairo, Egypt; 3 Department of Basic Medical and Dental Sciences, Faculty of Dentistry, Zarqa University, Zarqa, Jordan; 4 Pharmaceutical Chemistry Department, Faculty of Pharmacy, Sinai University (Arish Branch), ElArich, Egypt

**Keywords:** Pyroptosis, Pyroptotic inducers, Metalorganic, Polypeptide, Benzenoid, Heterocyclic

## Abstract

Pyroptosis, as a lytic-inflammatory type of programmed cell death, has garnered considerable attention due to its role in cancer chemotherapy and many inflammatory diseases. This review will discuss the biochemical classification of pyroptotic inducers according to their chemical structure, pyroptotic mechanism, and cancer type of these targets. A structure-activity relationship study on pyroptotic inducers is revealed based on the surveyed pyroptotic inducer chemotherapeutics. The shared features in the chemical structures of current pyroptotic inducer agents were displayed, including an essential cyclic head, a vital linker, and a hydrophilic tail that is significant for π-π interactions and hydrogen bonding. The presented structural features will open the way to design new hybridized classes or scaffolds as potent pyroptotic inducers in the future, which may represent a solution to the apoptotic-resistance dilemma along with synergistic chemotherapeutic advantage.

**Graphical Abstract:**

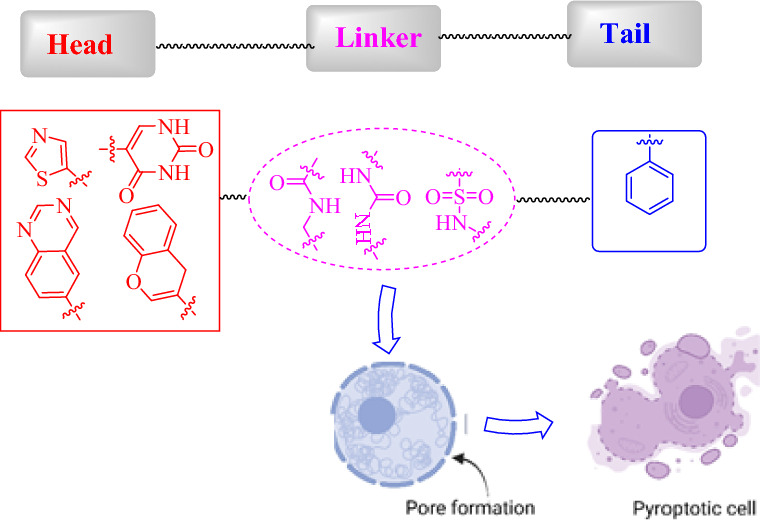

## Introduction

**Fig. 1:**
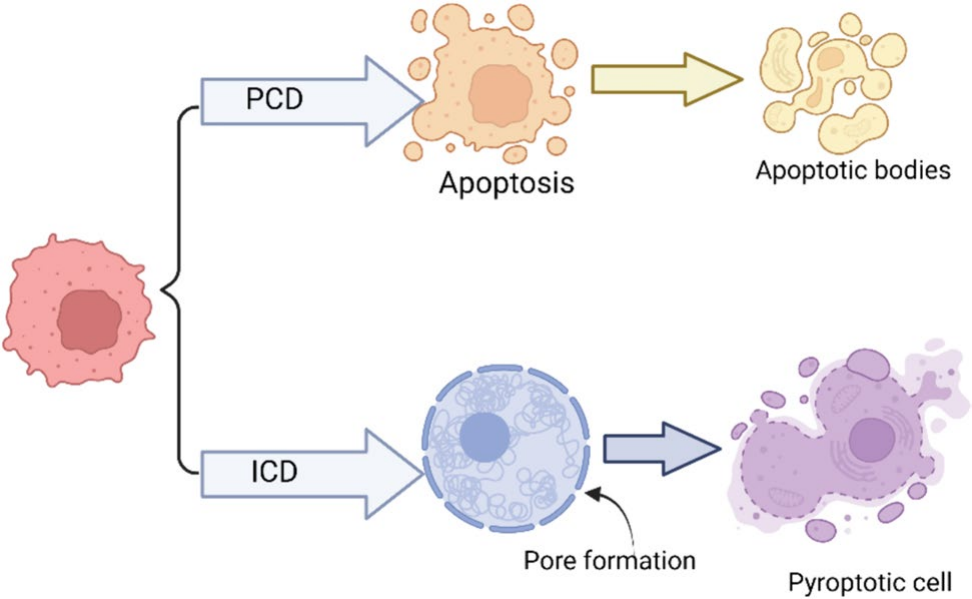
Morphological status in apoptosis and pyroptosis. *ICD* immunological cell death, *PCD* programmed cell death

Programmed cell death (PCD) plays a vital role in processes like morphogenesis, maintenance of homeostasis, and various diseases, notably cancer [1–3]. Morphologically, PCD can be categorized into inflammatory and non-inflammatory types. Apoptosis, a non-inflammatory type, involves no release of pro-inflammatory factors. Conversely, pyroptosis, along with other forms like necroptosis, etoptosis, netoptosis, ferroptosis, etc., are inflammatory types marked by the significant release of pro-inflammatory factors [4–6]. Pyroptosis, characterized by a bubble-like morphology [7] (Fig. 1), derives its name from the Greek word’s “pyro” meaning fire and “ptosis” meaning death [8]. Initially discovered in the immune defense against pathogens [9], pyroptosis is now implicated in various inflammatory diseases such as atherosclerosis [10–13], diabetic cardiomyopathy [14–16], Parkinson’s disease [17], multiple sclerosis [18], AIDS [19], and oncology [20–22]. Given the resilience of cancer, recent research has increasingly focused on exploring the connection between pyroptosis and cancer [23].

## Mechanisms of pyroptosis

In 1992, the Zychlinsky group made a groundbreaking discovery in the field of programmed cell death (PCD) by identifying pyroptosis while studying macrophages treated with *Shigella flexneri* [24]. Initially, this phenomenon was considered a subtype of apoptosis. Boise and Collins later distinguished between pyroptosis and apoptosis by subjecting macrophages to *Salmonella typhi*, identifying two key features: the release of inflammasomes and a reliance on caspase-1 rather than caspase-3, which is characteristic of apoptosis [25, 26]. However, differentiating between necroptosis and pyroptosis proved challenging.

Over the past decade, advancements in understanding inflammasomes and caspases, specifically caspases 1, 4, 5, and 11, have solidified their association with pyroptosis [27–31]. The mechanisms through which caspases initiate pyroptosis remained elusive until Feng Shao and his team discovered that gasdermins (GSDMs) play a crucial role in the initiation of the pyroptotic process by caspase-1 [32, 33]. Four major pyroptotic mechanisms have since been identified:

### Canonical pathway of pyroptosis

Inflammasomes play a pivotal role by orchestrating their assembly. This assembly process occurs concurrently with the cleavage of gasdermin D (GSDMD), leading to the subsequent release of interleukin-1β (IL-1β) and interleukin-18 (IL-18) [34, 35]. Inflammasomes are complex molecular structures activated when the host resists microbial infections, contributing to the development of adaptive immune responses. Additionally, inflammasomes are implicated in non-microbial diseases, and substantial evidence suggests their crucial involvement in oncogenesis, influencing processes such as proliferation, metastasis, and invasion. The assembly of inflammasomes commences with cytosolic pattern recognition receptors (PRRs), also known as inflammasome sensors. These receptors have the capability to recognize both pathogen-associated molecular patterns (PAMPs) and danger-associated molecular patterns (DAMPs). Upon activation of PRRs, downstream signaling pathways are initiated, leading to the generation of type I interferons and the release of pro-inflammatory cytokines [36, 37].

### Non-canonical pathway of pyroptosis

Involving human caspases 4, 5, and 11, the absence of upstream sensory complexes is notable. Instead, these caspases can be activated directly through interaction with intracellular lipopolysaccharide (LPS) via their N-terminal CARD domain. Notably, phospholipid-1-palmitoyl-2-arachidonoyl-sn-glycero-3-phosphorylcholine (oxPAPC), a TLR4 agonist, forms a complex with LPS and binds to caspases 4 and 11. This interaction results in a reduction of non-canonical inflammasome activation in macrophages, but this effect is not observed in dendritic cells. Activated caspases 4, 5, and 11 also have the capability to cleave gasdermin D (GSDMD) into its *N*-terminal fragment (N-GSDMD). Subsequently, N-GSDMD undergoes oligomerization and translocates to the cell membrane, forming pores on the plasma membrane. It is important to note that caspases 4, 5, and 11 do not cleave pro-IL-1β or pro-IL-18. However, they play a role in the maturation and release of IL-1β and IL-18 by participating in the NLRP3/caspase-1 pathway in specific cell types. Furthermore, the cleavage of GSDMD by caspases 4, 5, and 11 initiates the efflux of potassium ions (K^+^), triggering the assembly of the NLRP3 inflammasome and ultimately leading to pyroptosis [38–40].

### Caspase-3/8-mediated pathway

Members of the gasdermin protein family exhibit remarkable structural similarity. All gasdermins, except for DFNB59, feature both *C*-terminal and *N*-terminal domains, with the *N*-terminus playing a crucial role in executing pyroptosis. Initially, it was believed that caspases associated with apoptosis, such as caspase-3 and caspase-8, could not activate gasdermins to induce pyroptosis. However, recent research has revealed that specific chemotherapeutic drugs can induce caspase-3-mediated cleavage of gasdermin E (GSDME), particularly in cases with elevated GSDME expression. This cleavage leads to the generation of N-GSDME fragments, ultimately triggering pyroptosis in tumor cells [41–44].

### Granzyme-mediated pathway

CAR T cells rapidly activate caspase-3 in target cells by releasing granzyme B (GzmB). This activation initiates the caspase-3/GSDME-mediated pyroptotic pathway, causing extensive pyroptosis. More recently, researchers discovered that GzmB directly cleaves GSDME, inducing pyroptosis and further activating the antitumor immune response, leading to the inhibition of tumor growth. Subsequently, it was reported that natural killer cells and cytotoxic T lymphocytes (CTLs) induce pyroptosis in GSDMB-positive cells, contributing to the antitumor immune response [41, 45–49].

## The role of pyroptosis in cancer chemotherapy

Non-apoptotic cell death, including pyroptosis, represents a promising strategy for cancer treatment that is currently in the early stages of exploration in biomedical research [50]. Pyroptosis is frequently observed in tumor cells treated with conventional chemotherapeutic agents or emerging small molecule targets [51]. However, this induction occurs in both cancerous and normal cells, lacking specificity. There is a potential for enhanced benefits to patients if pyroptosis could be selectively induced in cancerous cells, thereby activating the immune response exclusively within the cancerous microenvironments [9].

## The role of pyroptosis in cancer immunotherapy

The primary goal of immunotherapy is to boost the body’s innate defenses for effective cancer combat. Pyroptosis, in this context, can activate macrophages and helper T cells (THs) within the tumor microenvironment, guiding them to eliminate dysfunctional tumor cells. Consequently, pyroptosis not only triggers an immune response against tumor cells but also enhances their immunogenicity. The immunogenic induction function of pyroptosis plays a pivotal role in overcoming the resistance that tumor cells often develop against the body’s immune system.

Ongoing studies on dendritic cell-based vaccines, immune checkpoint inhibitors, and adoptive T-cell therapies underscore researchers’ efforts to comprehend the precise role of immunogenic cell deaths (ICDs), particularly pyroptosis, in cancer immunotherapy [52, 53]. Investigations into cells undergoing pyroptotic cell death suggest that the release of inflammatory interleukins (specifically IL-1β and IL-18) and various damage-associated molecular patterns (DAMPs) during pyroptosis triggers the differentiation of multiple T helper cell subsets, including TH1 and TH17 cells. Furthermore, IL-18 exhibits a dual role in antitumor immune responses. It suppresses immune responses against tumors, particularly those involving M1 macrophages, natural killer (NK) cells, and CD8 T cells, while also promoting the release of IFN-γ and assisting cytotoxic T cells in their mission to eliminate tumor cells [54–56].

## Chemotherapeutic agents act as pyroptotic inducers

Chemotherapy remains the conventional treatment for various cancer types, initially showing positive responses. However, the development of resistance inevitably occurs, leading to the unfortunate outcome where most patients succumb to cancers that have become resistant to chemotherapy. Therefore, the investigation of chemotherapeutic drugs inducing pyroptosis in tumor cells holds significant importance [57], as well as exploring combinations of chemotherapeutic agents [58–60].

Drug repurposing involves exploring existing drugs for new therapeutic applications beyond their initially intended use. This strategy utilizes the knowledge and safety profiles of established medications to address different medical conditions or diseases. Rather than creating entirely new drugs, repurposing aims to find novel and effective uses for drugs that have already undergone extensive testing and approval processes. This approach can expedite the development of treatments and potentially reduce the cost and time required to bring new therapies to the market. In this review, pyroptotic inducers are categorized based on their chemical structure, with two main groups identified: acyclic and aryl-containing compounds. These primary categories will be further subdivided into additional subcategories.

## Acyclic pyroptotic inducers

This class of pyroptotic inducers can be subdivided into aliphatic, organometallic, or polypeptides drugs.

### Aliphatic pyroptotic inducers

**Fig. 2:**
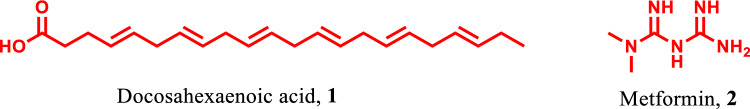
Pyroptotic inducers with aliphatic scaffold

DHA 1 is a conjugated polyene fatty acid that has demonstrated its pyroptotic action on breast cancer cells through caspase-1/GSDMD activation [61]. Metformin 2, a guanidine compound, has been identified as a pyroptotic agent in the treatment of various gastrointestinal (GIT) cancers, including human esophageal carcinoma cells [62], hepatocellular carcinoma [63], and other types [64]. This effect is typically achieved through the activation of the AMPK/SIRT1/NF-κB pathway and the induction of mitochondrial dysfunction, ultimately driving caspase-3/GSDME-mediated cancer cell pyroptosis (Fig. 2).

### Organometallic pyroptotic inducers

**Fig. 3:**
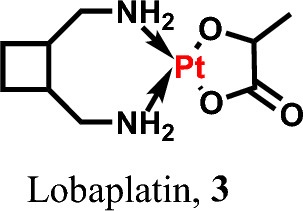
Pyroptotic inducers with organometallic scaffold

Lobaplatin 3, a second-generation platinum metallodrug, was originally synthesized and developed by ASTA Pharma in Germany in 1990, initially known by the research designation D-19466 [65]. Recently, Chen et al. discovered the role of lobaplatin in the treatment of cervical cancer cells by inducing pyroptosis via caspase-3/GSDME activation [66]. Lobaplatin 3 has been indicated for the treatment of various cancer types as a pyroptotic inducer [67] (see Fig. 3)

### Polypeptide pyroptotic inducers

**Fig. 4:**
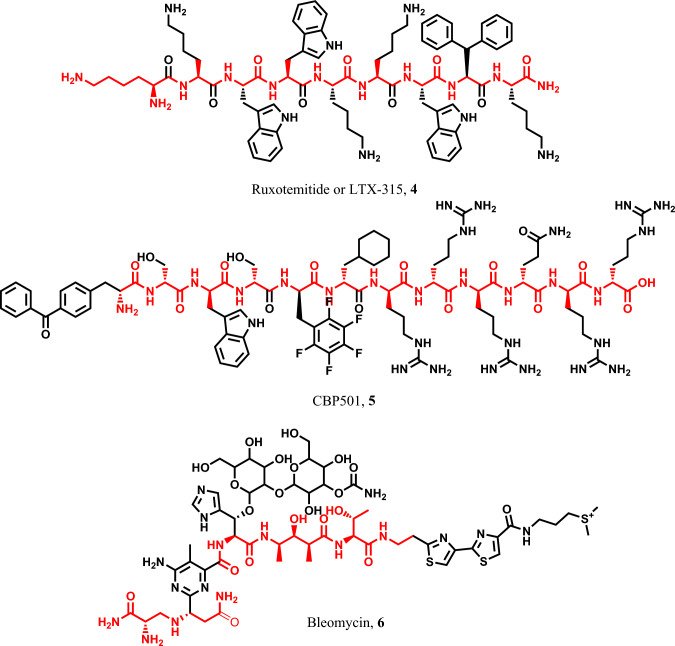
Pyroptotic inducers with polypeptide scaffold

Ruxotemitide, also known as LTX-315 **4**, is a newly designed peptide consisting of 9 amino acids, inspired by the structure of the host defense peptide bovine lactoferrin [68, 69]. It has recently emerged as a treatment option for various cancer types, functioning as a pyroptotic inducer through multiple pyroptotic pathways [70, 71]. CBP501 **5**, on the other hand, is a 12 amino acid peptide known for its ability to disrupt the G2 checkpoint and modulate calmodulin. This peptide enhances the influx of platinum into tumor cells and triggers pyroptosis in cancer cells [72–74]. Bleomycin **6** is a conjugated heterocycle with long-chain peptide employed in the treatment of several cancer types, including Hodgkin’s lymphoma, non-Hodgkin’s lymphoma, testicular cancer, ovarian cancer, cervical cancer, and others [75–77] (Fig. 4).

## Aryl-containing pyroptotic inducers

### Benzenoid pyroptotic inducers

**Fig. 5:**
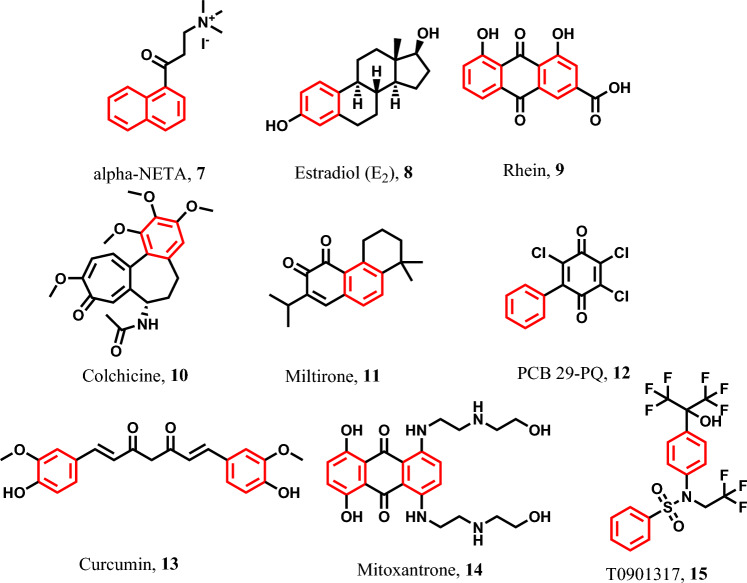
Pyroptotic inducers with benzenoid scaffold

Alpha-NETA **7** was observed to induce pyroptosis in ovarian cancer cells through the activation of caspase-4 and GSDMD, as indicated by studies [78–81]. Furthermore, Estradiol (E2) **8** has demonstrated pyroptotic activity on hepatocellular carcinoma (HCC) tumor cells and others [82–85] (Fig. 5). In addition, Rhein **9** has exhibited pyroptotic activity in colorectal cancer (CRC) through the activation of caspase-1 and GSDME [86]. Moreover, Colchicine **10** has been reported to induce pyroptosis in pancreatic cancer cells [87] and it has also demonstrated pyroptotic effects in numerous other cancer cell types [88]. Also, Miltirone **11** is a chromene derivative for its pyroptotic activity in HCC and others [83, 89, 90]. PCB 29-PQ **12** has been reported to exhibit pyroptotic activity in cervical cancer cells by activating caspase-1 and GSDMD [81] (Fig. 5).

Additionally, Curcumin **13** is utilized in the treatment of acute leukemia, prostate cancer, colorectal cancer, and various other cancer types [91–94]. Mitoxantrone **14**, a fused polycyclic aryl compound, induces pyroptosis in breast cancer cells through the activation of caspase-1 and caspase-3, leading to GSDME activation [95–97]. However, T0901317 **15** a sulphonamide compound exerts its pyroptotic effects on non-small cell lung cancer (NSCLC) tumor cells by activating caspase-1 and NLRP3 [98–100] (Fig. 5).

### Heterocyclic pyroptotic inducers

#### Four-membered heterocyclic pyroptotic inducers

Paclitaxel, also known as Taxol **16**, which is an oxetane-containing structure, has been shown to induce pyroptosis in non-small cell lung cancer (NSCLC) tumor cells [35]. It also exhibits pyroptotic effects in various other cancer types, including ovarian cancer cells [101], lung carcinoma [102], pancreatic cancer [67], and many more.

**Fig. 6:**
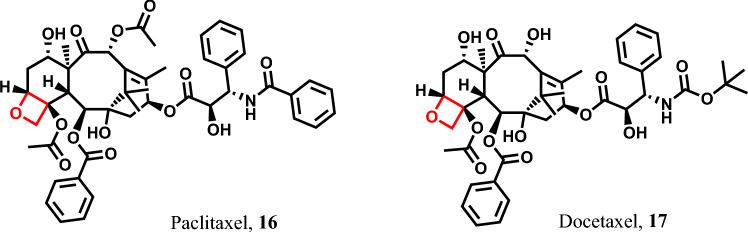
Pyroptotic inducers with four-membered heterocyclic scaffolds

Similarly, Docetaxel **17** demonstrates its pyroptotic action in head and neck squamous cell carcinoma tumor cells by activating caspase-3 and GSDME [103]. It has also been reported to induce pyroptosis in hepatocellular carcinoma [104], pancreatic adenocarcinoma [105], melanoma [42], and numerous other cancer types (Fig. 6).

#### Five-membered heterocyclic pyroptotic inducers

**Fig. 7:**
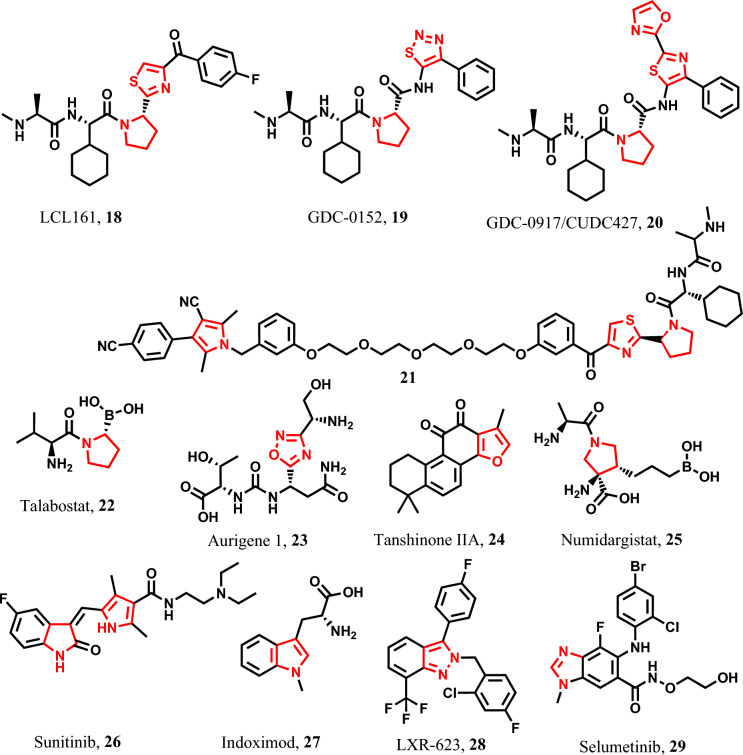
Pyroptotic inducers with five-membered heterocyclic scaffold

LCL161 **18** is a pyrrolidine-thiazole-containing compound that triggers pyroptosis in pancreatic cancer, leukemia, and multiple myeloma cells by activating caspase-1 and GSDMD [106, 107]. In addition, GDC-0152 **19** and GDC-0917 **20** are pyrrolidine-thiazole and pyrrolidine-oxazole-thiazole-containing compounds that target inhibitors of apoptotic proteins (IAPs), which are responsible for the resistance of tumor cells to apoptosis [108–111]. Furthermore, compound **21** is a hybrid structure, combining SMAC mimetic and anti-androgenic pharmacophores connected by a polyethylene linker. It induces pyroptosis in prostate cancer cells through the activation of caspase-1 [112]. Talabostat **22** is pyrrolidine derivative that triggers pyroptosis in acute myeloid leukemia cells by activating caspase-1 and GSDME [113, 114] (Fig. 7).

Moreover, aurigene 1 or AUPM 170 **23** an oxadiazole-containing anti-neoplastic agent is employed in lung carcinoma and prompts pyroptosis in cancer cells through the activation of caspase-1 and GSDMD [115]. However, tanshinone IIA **24** a furan-containing compound exhibits pyroptotic activity in cervical cancer cells by activating GSDMD [116] (Fig. 7). Also, numidargistat **25** is reported as a pyroptotic agent against breast cancer and various other cancers by activating GSDME, caspase-1, and caspase-3/GSDMD [117, 118] (Fig. 7).

On the other hand, sunitinib **26** acts as a PLK1 kinase inhibitor and induces pyroptosis in esophageal squamous cell carcinoma (ESCC) [119]. It also exhibits similar pyroptotic effects on hepatocellular carcinoma [120, 121]. Additionally, a methylated tryptophan compound known as indoximod **27** possesses immune checkpoint inhibitory properties and is employed in the therapy of various solid tumors [122, 123]. Furthermore, LXR-623 **28**, which is used in colon cancer treatment, functions as a liver X receptor inducer, inducing pyroptosis through caspase-1 and GSDME activation [124]. MAPK/ERK kinase inhibitor known as selumetinib **29** induces pyroptosis in pancreatic cancer cells [125, 126].

#### Hybrid five- and six-membered heterocyclic pyroptotic inducers

Dabrafenib **30** is a medication that selectively inhibits mutated forms of BRAF kinase and is employed either alone or in combination with trametinib **78** for the treatment of metastatic carcinoma [127, 128]. Dasatinib **31** is a PLK1 kinase inhibitor that enhances pyroptosis in lung carcinoma and neuroblastoma through GSDME activation [129–131].

**Fig. 8:**
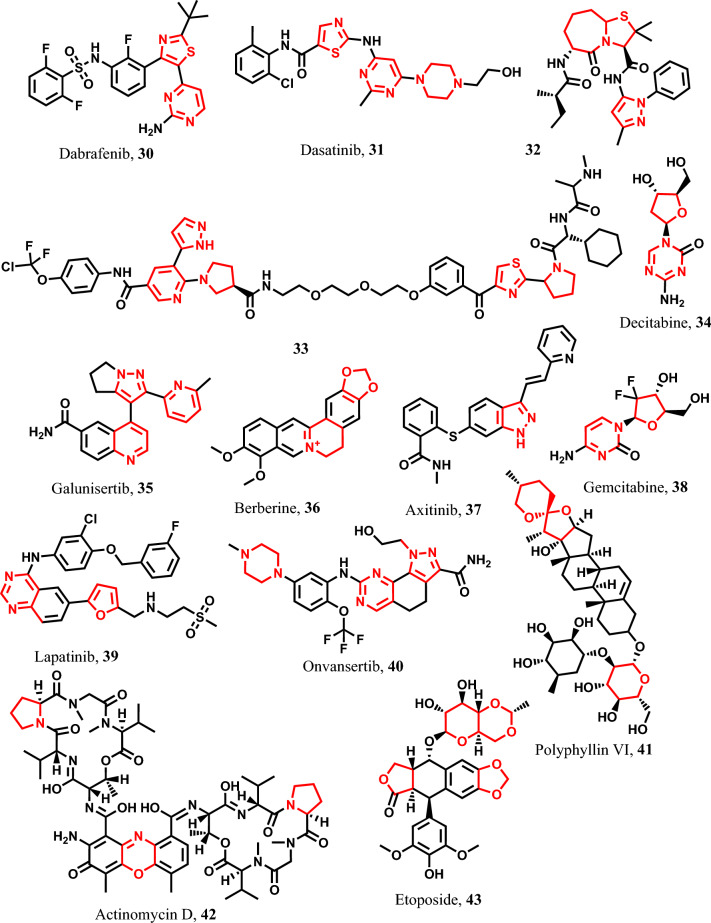
Pyroptotic inducers with hybrid five- and six-membered heterocyclic scaffold

Similarly, compound **32** is a second mitochondrial activator of caspases (SMAC) mimetic and represents a thiazole-based agent that induces pyroptosis in melanoma cells via caspase-3,7,9 activation [132, 133]. Furthermore, compound **33** combines SMAC mimetic and anti-androgenic elements through a polyethlenic linker and induces pyroptosis in prostate cancer cells via caspase-1 activation [112, 134] (Fig. 8). Additionally, decitabine **34** is a cytidine antimetabolite analog with potential anti-neoplastic activity [135, 136]. Moreover, galunisertib **35** triggers the pyroptotic pathway in glioblastoma, pancreatic cancer, and hepatocellular carcinoma through caspase-1/GSDMD activation [137, 138]. Berberine **36**, a natural quaternary ammonium salt derived from isoquinoline alkaloids, exhibits pyroptosis-inducing activity against HCC through caspase-1,3/GSDMD and E activation [139, 140]. Furthermore, axitinib **37** serves as a pyroptotic agents in colon adenocarcinoma through caspase-1/GSDMD activation and pancreatic adenocarcinoma through caspase-3/GSDMC activation [141–143]. Gemcitabine **38** is used in treatment of pancreatic carcinoma, HCC, and others through pyroptosis pathway [67, 144–146]. Lapatinib **39** and onvansertib **40** are PLK1 kinase inhibitors that improve the cisplatin response via inducing pyroptosis in ESCC and others through caspase-3/GSDME/Bax activation [22, 147–151]. Polyphyllin VI **41** is naturally related compound that has pyroptotic activity on many cancer cells [34, 42, 152, 153]. Similarly, actinomycin D **42** used in pyroptosis induction mediated through caspase-1,3/ GSDMD and GSDME activation [154]. Finally, etoposide **43** exerts its pyroptotic effects on various tumor cells by activating GSDME [155, 156] (Fig. 8).

#### Six-membered heterocyclic pyroptotic inducers

##### Pyridine-containing pyroptotic inducers

**Fig. 9:**
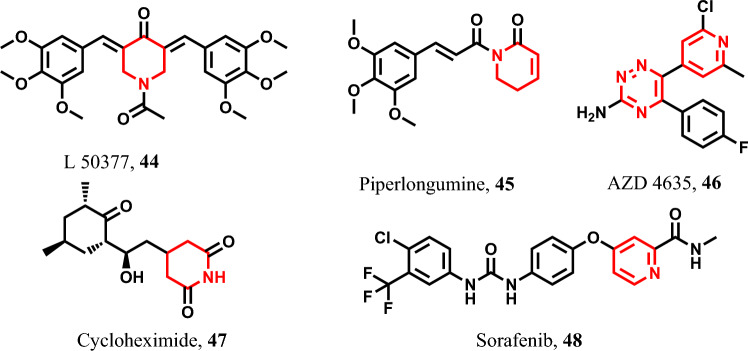
Pyroptotic inducers with pyridines and its hydrated derivative-containing scaffold

L 50377 **44**, piperlongumine **45**, and AZD 4635 **46** are pyridine-containing molecules with pyroptotic activities that exert their effects on non-small cell lung cancer (NSCLC) by activating GSDME [157, 158]. In addition, cycloheximide **47** has been reported as a pyroptotic agent against breast cancer and various other cancers [159]. Finally, sorafenib **48** is an oral multi-kinase inhibitor utilized in the treatment of hepatocellular carcinoma, thyroid cancer, and advanced renal carcinoma. Its pyroptotic activity has been observed in HCC and other tumor cells, primarily mediated by caspase-1 activation [42, 83, 120, 160] (Fig. 9).

##### Pyrimidine-containing pyroptotic inducers

**Fig. 10:**
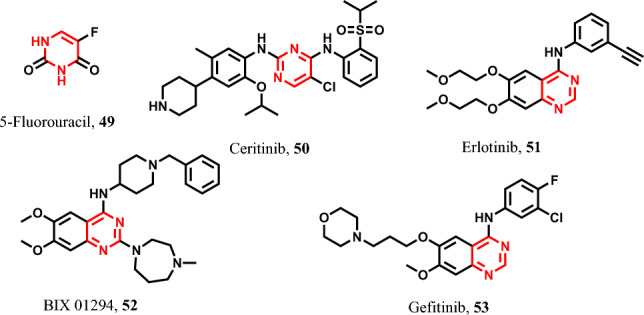
Pyroptotic inducers with pyrimidine-containing scaffold

5-Fluorouracil **49** induces pyroptosis in various cancer cells through the activation of GSDME [98, 161, 162]. However, ceritinib **50**, is utilized in the treatment of metastatic non-small cell lung cancer [163, 164] (Fig. 10). Furthermore, erlotinib **51**, featuring a quinazoline moiety, acts as a pyroptotic agent by initiating the pyroptosis process in lung and various other tumor cells through the stimulation of caspase-1, caspase-4, caspase-5, and caspase-11 [165, 166]. Also, BIX 01294 **52**, enhances the chemotherapeutic effect in gastric cancer types by inducing pyroptosis through the activation of caspase-3 and GSDME [167].

Finally, gefitinib **53** acts as a PLK1 kinase inhibitor, and it induces pyroptosis in esophageal squamous cell carcinoma (ESCC) and various other cancer types by triggering caspase-3, GSDME, and Bax activation [168] (Fig. 10).

##### Pyran-containing pyroptotic inducers

**Fig. 11:**
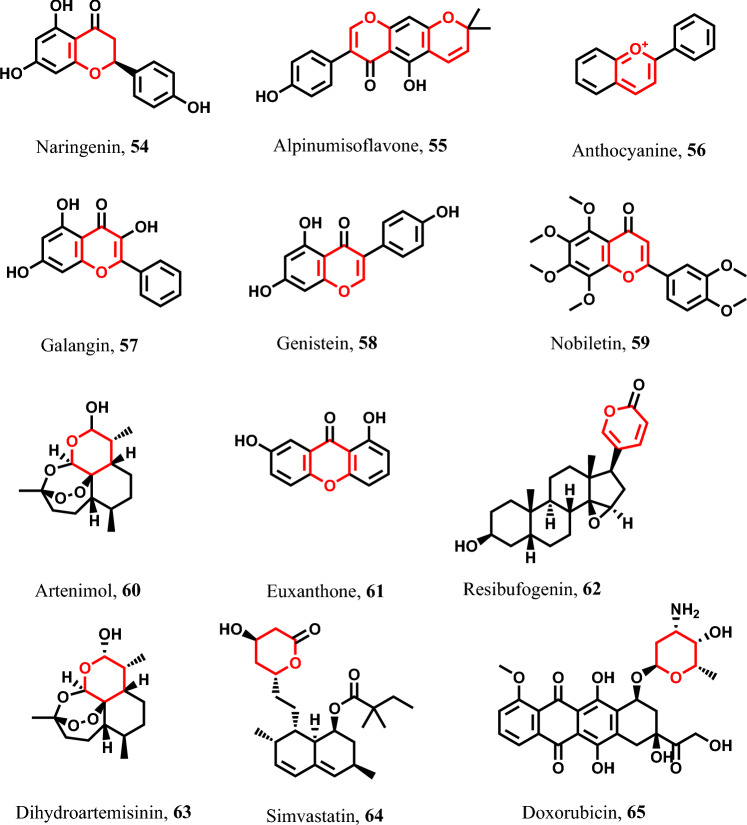
Pyroptotic inducers with pyran-containing scaffold

Various flavonoid subclasses displayed pyroptotic capabilities. For example, naringenin **54** is a natural flavanone used against HCC through caspase-1 activation [169]. Similarly, alpinumisoflavone **55** is used in ESCC treatment [169]. Anthocyanine **56** is a natural pigment used in OSCC through the activation of caspase-1/GSDMD/NLRP3 [170] (Fig. 11). Furthermore, galangin **57** is a potential anticancer agent against lung cancer, HCC, breast cancer, ovarian cancer, gastric cancer, colorectal cancer, retinoblastoma, and osteosarcoma exerting a pyroptotic action via inducing GSDME. Additionally, genistein **58** is an isoflavone derivative that has inhibitory activity against tyrosine kinase enzyme. It was found that genistein has pyroptotic activity against cervical cancer through the activation of caspase-8/GSDMC [171]. Also, nobiletin **59**, a methoxy flavone, has pyroptotic induction efficacy against breast cancer through caspase-1/GSDMD/NLRP3 activation [172]. Artenimol **60** is a sesquiterpene that displayed an anticancer activity against NSCLC with pyroptotic induction via GSDME activation [173], while euxanthone **61** has pyroptotic activity on HCC tumor cells by activating caspase-2 [35, 120]. Also, resibufogenin **62** exerts its pyroptotic activity on NSCLC tumor cells by activation of caspase-1/NLRP3 [89, 152]. Dihydroartemisinin **63** exerts its pyroptotic activity on PGC-1α tumor cells by activation of caspase-3 [174–177]. Similarly, simvastatin **64** exerts its pyroptotic activity on tumor cells by activation of caspase-1/NLRP3 pathway [175–178]. Finally, doxorubicin **65** displayed their pyroptotic action on melanoma tumor cells via caspase-3/GSDME activation [179, 180] (Fig. 11).

##### Miscellaneous six-membered heterocyclic pyroptotic inducers

**Fig. 12:**
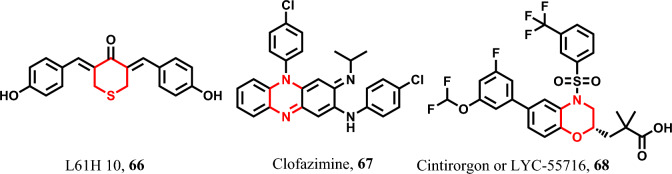
Pyroptotic inducers with miscellaneous six-membered heterocyclic scaffold

L61H 10 **66** is a naturally occurring compound with demonstrated pyroptotic activity against lung cancer cells. Its mode of action involves the activation of caspase-3/GSDME [181]. Moreover, clofazimine **67** has more recently been found to induce apoptosis in the treatment of various solid cancers such as HCC and cervical carcinoma [182]. In addition, cintirorgon **68** also known as LYC-55716 serves as a pyroptotic agent to enhance pyroptosis in gastric and esophageal tumors. Its mechanism involves the activation of caspase-3/GSDME [183, 184] (Fig. 12).

#### Fused heterocyclic pyroptotic inducers

**Fig. 13:**
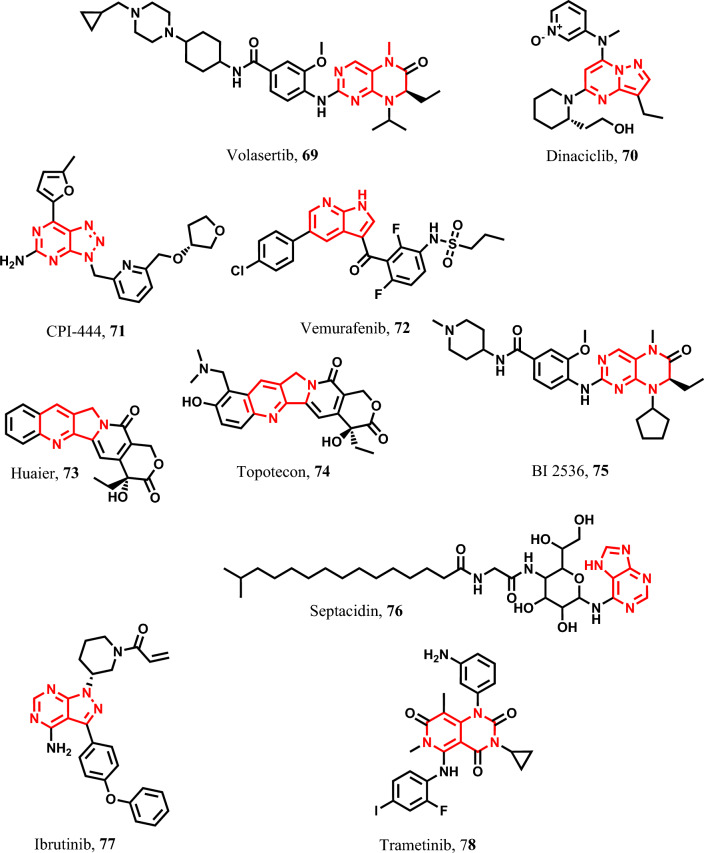
Pyroptotic inducers with fused heterocyclic scaffold

Furthermore, volasertib **69**, shown in Fig. 13, is a PLK1 kinase inhibitor that induces pyroptosis in acute myeloid leukemia and many other tumors through caspase-3/GSDME/Bax activation [151, 185]. Dinaciclib **70** is a selective inhibitor of cyclin-dependent kinases (CDKs), CDK1,2,5,9, used to induce pyroptosis in melanoma through caspase-1/GSDME/Bax activation [186]. CPI-444 **71** is an immune checkpoint inhibitor used as a pyroptotic agent in non-small-cell lung carcinoma (NSCLC) and MM via caspase-1/GSDMD activation [187]. Furthermore, vemurafenib **72** is a BRAF kinase inhibitor used to induce pyroptosis in melanoma through caspase-1/GSDME activation [188]. Huaier **73** is used in the treatment of numerous types of solid cancers such as NSCLC. Additionally, topotecan **74** is a semisynthetic derivative of camptothecin with topoisomerase I inhibitory activity used to treat many solid tumors, such as gastric carcinoma and ESCC. It exerts its pyroptotic activity by caspase-3/GSDMD and E activation [159]. BI 2536 75 is also used to induce pyroptosis in GIT cancers such as colorectal cancer via caspase-3/GSDME activation. In colorectal cancer, BI 2536 **75** is used to induce pyroptosis via caspase-3/GSDME activation [189]. Furthermore, septacidin **76** is used as pyroptotic agent in colon adenocarcinoma through caspase-1/GSDMD activation [190]. Ibrutinib **77** is a specific Bruton’s tyrosine kinase inhibitor used as a pyroptotic agent in chronic lymphocytic leukemia, HCC, and lung adenocarcinoma through caspase-3/GSDME activation [191–193]. Likewise, trametinib 7**8** is a dual kinase inhibitor used to induce the pyroptotic pathways in pancreatic cancer and melanoma through caspase-1/GSDMD activation [42, 194, 195] (Fig. 13).

## Conclusion and future directions

Chemotherapeutic agents usually suppress tumor growth by inducing apoptosis in cancer cells. However, cancer cells develop resistance against anticancer agents by evading apoptosis. So, it is necessary to explore nonapoptotic pathways to suppress the growth of cancer cells.

**Fig. 14:**
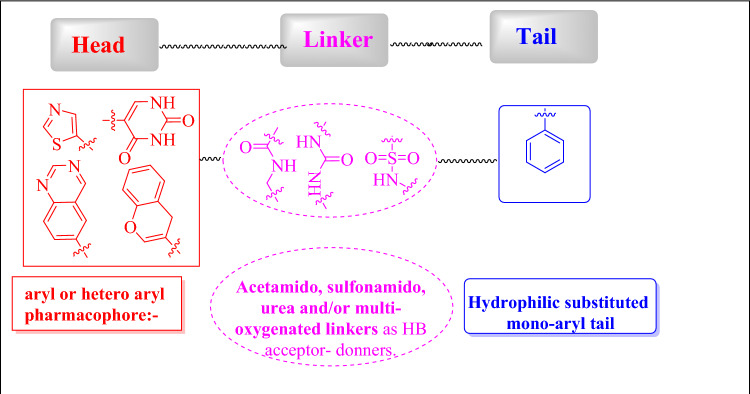
SAR elaboration of promising anticancer pyroptotic agents

Although pyroptosis garnered greater biomedical research attention, the drug design strategies of pyroptotic active agents are still preliminary. Therefore, some structural fingerprints of pyroptotic agents were scrutinized in the present review. We explored the concept of pyroptosis from a biological perspective with chemical views. In terms of SAR analysis, pyroptosis can provide an immune-stimulatory response in the tumor microenvironment, thus increasing cancer immunotherapy efficacy. Despite the considerable efforts in the field of synthetic medicinal chemistry for new anticancer agents, designing and testing selective pyroptotic agents are still warranted. From the structural screening of the surveyed pyroptotic inducers, we propose the following traits of a lead pyroptotic inducer molecule with the main characteristic features depicted in Fig. 14. The proposed lead molecule may be built from three major parts: head, linker, and tail.

In summary, the proposed lead pyroptotic inducer molecule comprises a mono or bicyclic head with a 5- or 6-membered aryl or heteroaryl pharmacophore, exemplified by compounds like dasatinib. The inclusion of sulfonamide, acetamido, urea, and/or multi-oxygenated linkers is considered, providing hydrogen bond acceptor–donor functionalities. Additionally, a substituted mono-aryl system serves as the tail, contributing to *π*−*π* hydrophobic interactions. Our objective is that this structure–activity relationship (SAR) study can contribute to the development of future pyroptotic inducer agents.

## Data Availability

No datasets were generated or analyzed during the current study.
